# Profile and clinical characterization of seizures in hospitalized children

**DOI:** 10.11604/pamj.2016.24.313.9275

**Published:** 2016-08-16

**Authors:** Ernestina Ernest Mwipopo, Shahnawaz Akhatar, Panpan Fan, Dongchi Zhao

**Affiliations:** 1 Dongchi Zhao, Department of Pediatrics, Zhongnan Hospital of Wuhan University, Wuhan, Hubei, China

**Keywords:** Seizures, children, febrile seizures

## Abstract

**Introduction:**

Seizure is the commonest pediatric neurological disorder, which is frightening to caretakers. The current study aims to determine profile, clinical spectrum and analyze the commonest etiology of seizures in children admitted to a tertiary hospital in Central China.

**Methods:**

This was a hospital based retrospective study carried out in Zhongnan Hospital of Wuhan University, China. Computerized data was collected from January 2012 to May 2015. Variables collected were demographics, clinical presentations and laboratory tests; brain imaging studies, electroencephalography, diagnosis, prognosis, outcome and duration of hospitalization.

**Results:**

A total of 200 patients were admitted with seizures. There were 109 (54.5%) males and 91 (45.5%) females. Among these patients, 193 (96.5%) were aged 1 month to 5 years and 182 (91.0%) presented with seizures and fever. Generalized tonic-clonic seizure was the most common seizure type in 196 (98.0%) children. Febrile seizure was the leading etiology of seizure in 175 (87.5%) children followed by epilepsy in 11 (5.5%) children. There were only 3 (2%) children with central nervous system infections. Abnormal brain images were noted in 10 (20%) out of 50 patients. Among 193 children tested for different infections, 49 (25.4%) had positive results. Viral infections were commonest infections by 49.0%, atypical bacterial 34.7% and 16.3% coinfections.

**Conclusion:**

Seizure was the commonest neurological condition of children admitted in our hospital, febrile seizures being the commonest etiology. The prognosis and outcomes were good but there were prolonged days of hospitalization. Children with unprovoked seizures require brain-imaging studies for better understanding of seizure etiology.

## Introduction

Seizures are quite common worldwide, especially among children, and have a wide range of causes. They are the most common and frightening pediatric neurological disorder, with 4-10% of children experiencing at least one seizure within first 16 years of life. Children below 3 years of age have the highest incidence of seizures than older children [1]. Seizures may be benign and self-limited, requiring minimal treatment, or may be the initial sign of a serious medical illness. The common etiologies of seizures are determined by the geographical variations. Febrile seizures (FS) are reported in many studies to be the most common type of seizures seen in the pediatric population, and majority being less than five years of age [2]. Infections are the most associated causes and have a good outcome [3]. It affects 2% to 4% of all children in Europe and the United States by their fifth birthday [2]. The incidence in other areas varies from 0.35% in Hong Kong [4], 1% in China to more than 8% in Japan and 14% in Guam [2].

Even though FS are common in tropical countries, the prevalence of acute symptomatic seizures may be higher than Western countries and have a poorer outcome. In these countries, the incidence of acute seizures is higher and more febrile status epilepticus are seen, therefore the outcome is worse because the etiology is different [5]. In some Asian countries central nervous system infections (CNS) especially neurocysticercosis, are mentioned to be the main cause of seizures [6–8]. In Sub Saharan Africa malaria is the leading cause [5]. Therefore a major risk factor for cognitive, neurological impairment and development of epilepsy in children living in these regions is acute seizures [5, 9, 10]. In patients with simple FS routine brain imaging like head CT (cranial tomography) scan or MRI (magnetic resonance imaging), is discouraged as it has no additional diagnostic and prognostic value [9]; and there is a concern about the cost of these investigations in developing countries which are having limited and poor resources [1]. Nevertheless, children admitted with afebrile seizure are often evaluated using these imaging examinations even though some reports indicate they should not be routinely done in these patients [1]. Further investigations likewise, are not routinely suggested, unless necessary. Complete blood count is indicated in children who appear ill, electrolyte studies and serum glucose are considered when there is a history of vomiting or diarrhea and urinalysis can be done when the source of fever is not identified [11]. In this hospital based retrospective study, we analyzed profile, commonest etiology and clinical spectrum of seizures in children who were admitted to pediatrics ward with complaint of seizure.

## Methods

### Patient population

**Figure 1:**
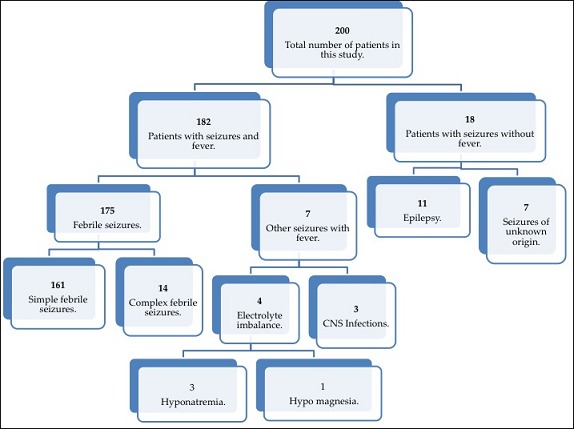
Flow chart describing patients involved in this study

This was a hospital based retrospective study, which focused on a period of 3 years and 5 months, from January, 2012 to May, 2015. We included 200 children with presenting complaint of seizures who were admitted at pediatrics department of Zhong Nan Hospital of Wuhan University. We excluded children with seizures above 14 years and neonatal seizures below 1 month of age. There were a total of 5749 children hospitalized during this study period. Study design is shown as Figure 1.

### Methods

The data extracted from the computerized medical records of each patient included: date of admission, age (range from 1 month to 14 years), sex, type of seizure, maximum temperature recorded during the illness, duration of convulsion, frequency of seizures in the current illness, duration of fever prior to the episode of seizure, past medical history, family history of seizure or epilepsy, developmental history, associated symptoms, duration of hospital stay, final diagnosis and outcome. Laboratory test results recorded included serum electrolytes, blood culture, blood glucose, cerebrospinal fluid (CSF) analysis, nasal and throat mucosa viral antigen tests, stool rotavirus antigen test, cytomegalovirus (CMV), Epstein Barr virus (EBV), Mycoplasma pneumoniae (MP) and Chlamydia pneumonia (Cpn) immunoglobulin (Ig M/Ig G) tests. Neuroimaging results of head CT scan or MRI and EEG (electroencephalography) findings were also recorded. Patients were divided into groups of seizure with fever and seizure without fever; those with temperature 37.6°C and above were the fever group while those with temperature below that were the group without fever. All children admitted in the pediatric ward had axillary temperature taken as a daily routine measurement. Type of seizures was classified as generalized or focal seizures. Patients were categorized into two age groups: age group of 1 month to 5 years and age above 5 years to 14 years. Status epilepticus was defined as, “a single epileptic seizure of more than 30 minutes or a series of epileptic seizures during which function is not regained between ictal events in a period more than 30 minutes long” [12]. FS definition was adopted from the International League against Epilepsy (ILAE) which is defined as “a seizure occurring in childhood after 1 month of age associated with a febrile illness not caused by CNS infection, without previous neonatal seizures or a previous unprovoked seizure, and not meeting the criteria for other acute symptomatic seizures” [12]. FS are classified as simple FS which are generalized in nature, lasting less than 15 minutes and do not recur within 24 hours and complex FS which are prolonged, lasting more than 15 minutes, occur more than once in 24 hours, or have focal features [13]. Other etiologies were taken as recorded from the final diagnosis but also based on the basis of recorded clinical findings and laboratory investigation and verified with standard reference.

### Data analysis

The data was analyzed using Statistical Package for the Social Sciences (SPSS) for Windows Version 20 (IBM, Chicago). Mean, median, mode, Chi-square test (χ^2^) or Fisher’s exact test was used to analyze data for continuous and categorical variables as appropriate. P value less than 0.05 was regarded as statistically significant.

### Ethical considerations and approval

This study involved the use of data from the computerized clinical records. Therefore it had no direct impact to patients, however all measures to protect privacy and confidentiality was maintained. No patient’s name was mentioned instead the Hospital Registration numbers were used. This was ensured as the study was done under supervision. Permission to perform this study was obtained from the Zhongnan Hospital authority.

## Results

### Demographic characteristics of children with seizures

**Figure 2:**
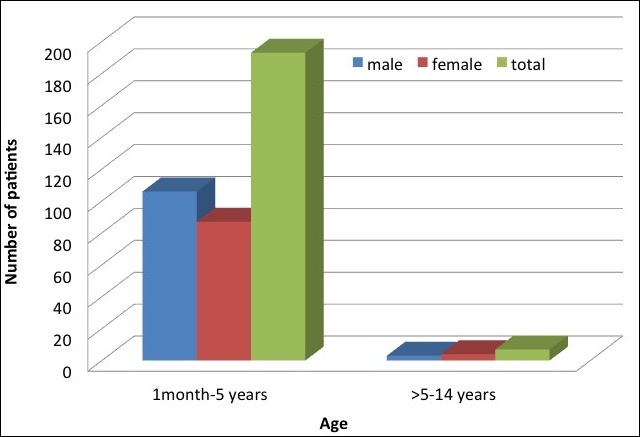
Age and sex distribution of children admitted with seizures

There were 200 (3.5%) children who were admitted with seizures as their presenting complaint, among a total of 5749 patients admitted to the pediatric ward; from 1 month to 14 years of age during the 3 years 5 months study period. Males were 109 (54.5%) and 91 (45.5%) females with male to female Ratio of 1.2:1. Among 200 children, 193 (96.5%) were in the age group 1 month to 5 years and only 7 (3.5%) were above 5 years (Figure 2). History of fever during this seizure illness was present in 182 (91.0%) children and 179 (92.7%) were in the age group 1month to 5 years. Among these 182 children, most of them 151 (83.0%) had a seizure within 24 hours of the onset of fever, whereby only 3 (1.6%) developed fever after the seizure had already occurred. Afebrile seizures (seizures without fever) were common 5 (57.1%) in age group 5 to 14 years. Generalized seizure mostly tonic clonic was the predominant type of seizures which was seen in 196 (98.0%) while 179 (98.4%) of them were febrile. Children with focal seizures were only 4 (2%) and 2 (50%) of them had status epilepticus. Family history of seizure related disorders was noted in only 19 (9.5%) patients (Table 1).

**Table 1:** Demographic characteristics of patients with seizures with and without fever

	No feverN=18(%)	FeverN=182(%)	TotalN=200(%)	P value
**Sex**				0.555
Male	11(61.1)	98(53.8)	109(54.5)	
Female	7(38.9)	84(46.2)	91(45.5)	
**Age**				0.001^+^
1month-5years	14(77.8)	179(98.4)	193(96.5)	
>5-14years	4(22.2)	3(1.6)	7(3.5)	
**Type of seizure**				0.316
Generalized	17(94.4)	179(98.4)	196(98.0)	
Focal	1(5.6)	3(1.6)	4(2.0)	
Family history of FS/ Epilepsy	3(16.7)	16(8.8)	19(9.5)	0.388

+ Statistically significant by Fisher’s exact test. FS- Febrile seizure

### Investigation results and diagnosis in children with seizures

**Figure 3:**
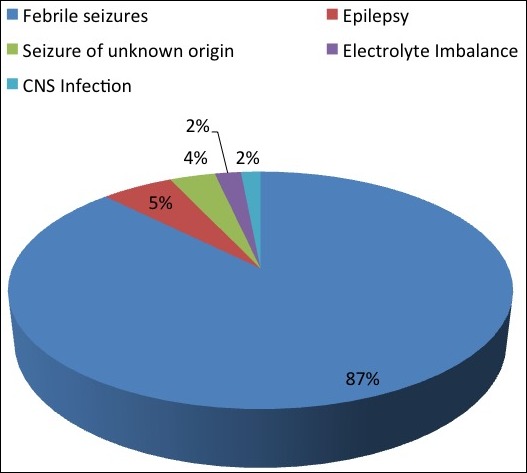
Etiology of children with seizures

Electroencephalogram (EEG) was reported in a total of 14 patients, while abnormal results were found in 11 (78.6%) children. **Table 2** above shows the findings of 6 children who had abnormal brain imaging results. The first child with brain atrophy had ataxia. There were a total of 10(20%) children with abnormal findings among 50 patients who had these investigations. Other 4 children had nasal sinusitis and/or mastoiditis on their imaging findings. We found out children with afebrile seizures had more brain imaging done (66.7%); and just 20.9% of children with fever and seizures had these investigations. Most of children with seizures in this hospital had FS, therefore only one lumber puncture was done during the study period. A total of 52 blood cultures were done and no bacterial growth was found in any sample. Several infectious agents’ antigen tests were done to patients with history of fever to determine the etiology of fever with seizures. Stool rotavirus (RV) antigen tests were performed in patients with watery diarrhea. Nasal and throat mucosa specimens were taken to determine respiratory tract viruses in all patients with respiratory symptoms. The 7 respiratory viral antigen tests included respiratory syncytial virus (RSV), adenovirus, influenza A & B, parainfluenza I, II and III. Cytomegalovirus (CMV), Epstein Barr virus (EBV) and atypical bacteria (MP- Mycoplasma pneumoniae & Cpn- Chlamydia pneumoniae) immunoglobulin (Ig M/Ig G) tests were done from blood serum of patients. Among 193 children whom different infectious agents were tested, 49 (25.4%) had positive test results. There were a total of 24 (49.0%) patients with viral infections, 17 (34.7%) with atypical bacterial infections and 8 (16.3%) with coinfection (viral & atypical bacteria). Distribution of infections (Table 3) was significantly associated with the seasons of the year with more than half of all infections (59.2%) found during winter season. FS was the commonest diagnosis in 175 children, followed by epilepsy in 11 children. Single unprovoked seizure (SUS) included children with a single/ isolated seizure (Figure 3). Patients with electrolyte imbalance had either hyponatremia or hypo magnesia. Those with CNS infection had viral encephalitis. Patients were discharged from the hospital when afebrile, after successful treatment. The general outcome was good and there was no death among patients with seizures within the study period. Children stayed in hospital for duration of 1 day to 2 weeks. The mean duration of hospital stay was 6 days. The mode was 5 and 6 days. Patients with positive infection tests stayed longer in hospital (more than a week) by 32.7% comparing to children without infection, whereby only 16% of them were admitted for more than 1 week (p value 0.012).

**Table 2:** Details of abnormal brain imaging studies and seizure causes in 6 children

No.	Age/Years	Sex	Diagnosis	EEG	Type of imaging	Imaging findings
1.	1.5	M	FS	No	CT scan	Bilateral brain atrophy of parietal and occipital areas.
2.	1.5	F	FS	Abn	CT scan	Enlarged right lateral ventricle.
3.	7	F	SUO	Abn	CT scan	Right choroid plexus cyst.
4.	5	M	SUO	Abn	MRI	Abnormal left hippocampus.
5.	1	F	Epilepsy	No	CT scan	Meningeal inflammation.
6.	3	M	Epilepsy	No	MRI	Undeveloped corpus callosum.

Abn- Abnormal; CT- Cranial tomography; EEG- Electroencephalogram; FS- Febrile seizure; F- Female; MRI- Magnetic resonance imaging; M- Male; No- Not done; SUS (single unprovoked seizure).

**Table 3:** Distribution of infections according to the seasons of the year

Seasons	No infectionN=144(%)	InfectionN=49(%)	TotalN=193(%)	P value
Winter	54(37.5)	29(59.2)	83(43.0)	0.046^+^
Spring	23(16.0)	3(6.1)	26(13.5)	
Summer	40(27.8)	9(18.4)	49(25.4)	
Autumn	27(18.8)	8(16.3)	35(18.1)	

+ Statistically significant

### Clinical analysis in patients with febrile seizures

During the study period, FS were diagnosed in 175 (87.5%) of 200 patients with seizures, including 96 boys (54.9%) and 79 girls (45.1%). Out of these 175 patients, 161 (92.0%) had simple FS and 14 (8.0%) had complex FS type. The underlying diagnoses of these children with FS included: upper respiratory tract infections (URTI) 127 (72.6%), lower respiratory tract infections (LRTI) 29 (16.6%), acute gastroenteritis 12 (6.9%) and other infections 7 (4.0%). URTI involved infections like tonsillitis, pharyngitis, laryngitis, sinusitis, otitis media and the common cold whereby LRTI included bronchitis and pneumonia. Other infections included acute skin rashes and local wound infection. Only 8% of patients with FS (14 children) had family history of FS in this study, none had family history of epilepsy and were not significantly associated with the seizure recurrence. Recurrence of FS was observed in 54 (30.9%) patients and majority of them (83.3%) were aged above 18 months. We found that 83.9% of those aged 18 months or below had their first seizure. The study revealed that the likelihood of recurrence of FS was associated with age at first episode (p=0.04). There was no association with sex, family history of seizures, type of FS, seizure duration, duration of hospital stay, maximum temperature during this illness, duration interval between seizure and onset of fever and frequency of seizure with recurrence of FS. The current study showed maximum temperature during febrile illness had association with different factors which were statistically significant (**Table 4**).

**Table 4:** Association of patients’ clinical presentation with maximum temperature during the current febrile illness

	<39°CN=43(%)	≥39°CN=132(%)	TotalN=175(%)	P value
**Recurrence**				0.22
First seizure	49(74.2)	88(65.7)	137(68.5)	
recurrent	17(25.8)	46(34.3)	63(31.5)	
**Fever before seizure**				0.020^+^
≤12 hours	18(41.9)	31(23.5)	49(28.0)	
>12 hours	25(58.1)	101(76.5)	126(72.0)	
**Type of FS**				0.021^+^
Simple FS	36(83.7)	125(94.7)	161(92.0)	
Complex FS	7(16.3)	7(5.3)	14(8.0)	
**Duration of seizure**				0.014^+^
≤ 3 minutes	23(53.5)	35(26.5)	120(8.6)	
>3 minutes	20(46.5)	97(73.5)	55(31.4)	
Infections	31(72.1)			0.003^+^
Negative	3(7.0)	99(75.0)	130(74.3)	
Viral	5(11.6)	27(20.5)	30(17.1)	
Atypical bacteria	4(9.3)	3(2.3)	8(4.6)	
Mixed		3(2.3)	7(4.0)	

+ Statistically significant. FS-Febrile seizure.

## Discussion

Seizures have been found to have a higher incidence in younger children in many studies with a decreasing frequency in the older age group, and are found to be more common in males [1, 4, 6]. The same was observed in this study whereby most children were younger than 5 years and higher prevalence in males. But slightly higher prevalence in female was noted in the age group more than 5 years which was also a finding in a study done in Nepal by Adhikari et al [1]. Seizures were associated with fever in 91.0% of all cases and most of them (92.7%) were in age group 1 month to 5 years due to the fact that febrile seizures are commonest seizures in this age group. Apart from that 83.0% had a seizure within 24 hours of the onset of fever. Therefore a child who has not yet developed a seizure within 24 hours since onset of fever, is less likely to develop a seizure, and thus can help reduce parental fear of fever (fever phobia). Most studies [1, 5, 6] show generalized tonic-clonic seizures are more common when compared to partial seizures which was a similar finding in this study and was found to have higher incidence among febrile children. Partial seizures were rare, only 2.0% in the current study; unlike developing countries with high incidence of neurocysticercosis, where studies reported partial seizures were common [8]. Insufficient evidence is available to make a standard recommendation or guideline for the use of routine neuroimaging in children with first unprovoked seizure [10, 14]. In our study only a few EEG tests were done among the children with seizures because most patients did not agree to have this investigation. EEG is recommended as part of the neurodiagnostic evaluation of the child with an apparent first unprovoked seizure [15, 16]. It is not helpful in children with simple febrile seizures [17]; but are probably most helpful if there is doubt about whether FS has really occurred, because EEGs carried out on the day of the seizure are abnormal in as many as 88% of patients [18]. Routine neuroimaging after simple FS is discouraged and has no additional diagnostic or prognostic value [9, 13]. It is also not likely to be helpful in well -appearing children after first complex FS [9]. Nevertheless, in afebrile children with seizures routine neuroimaging is recommended [1]. In a study by Rasool A et al, found out that abnormal EEG increases the risk of having abnormal neuroimaging but also reported that normal EEG does not rule out having an abnormal neuroimaging. Therefore they concluded that clinical examination and EEG results are good indicators and can be used as one of the criteria for ordering neuroimaging in new-onset seizures, more so in partial than generalized seizure [19].

We found out that FS was the main etiology of seizures below five years and overall (87.5%) in our pediatrics department. In most other studies, FS were also reported to be the most common diagnosis in pediatric population and account for majority of seizures seen in children younger than 5 years of age [20–22]. Other seizures in this study were epilepsy (seizure disorder) 5.5%. CNS infections were the least, with only 1.5% of all children with seizures. Adhikari et al found out in overall, seizure disorder was the commonest etiology (33.6%), followed by febrile seizures (30.5%) and CNS infection 25.2% [1]. In different studies worldwide male accounted a higher percentage. Our study reemphasized as previous studies [4, 20, 23–25] that FS was more frequent in boys than girls. As this study was done in China; and China has a trend of having more males than females in the population, this may not necessarily indicate that males are more affected by females. On the other hand, it may be possible that male children are biologically more vulnerable to FS. Generally, simple FS (84% to 89%) is common than complex FS (11% to 16%) [4, 26, 27]. Likewise, majority of children in our study had simple FS (92.0%). Most children with FS, approximately 97%, never develop epilepsy, but the risk is increased compared to healthy controls [28, 29]. A history of FS is present in 10-15% of people with epilepsy or unprovoked seizure, which is several times higher than the 2-4% seen in the general population [30]. Following a simple FS, the risk of epilepsy is 1-2.4%, while following a complex FS it is increased to 4.1-6% [28]. This may explain the low incidence of complex FS in China as well as few epilepsy cases, comparing to tropical countries that have more complex FS resulting in more epilepsy cases.

According to one report, approximately 25-40% of children with FS have a positive family history [31]. In 2010, Chen CY et al [20] noted that family history of FS accounted for 10.5% of cases. In the current study, only 8% of the children had a family history of FS. However, there is a probability of parents to be unaware of their past episodes of FS because they occurred when they were young, and this may be a confounding factor to miss the exact family history. Family history of FS is a risk factor for recurrence as mentioned by several literatures [32, 33]. There was no significant association of FS recurrence with family history of FS in our study. Other important risk factors for recurrence in other studies were early age of onset and complex FS [4]. Both factors were not found to be statistically significant in this study, probably due to the small study size. More than 50% of children with FS had URTI in our study. URTI were also the commonest cause of fever in other studies [23, 25, 34, 35]. In tropical regions, malaria was the commonest cause of fever [34, 36]. Among 12 children who had diarrhea in this study and were diagnosed with gastroenteritis; 67% (8) had stool rotavirus antigen test positive. Likewise, there are studies which reported that diarrhea was a highly associated symptom in patients with seizures, and rotavirus infection was an identified etiology [20, 31, 37, 38]. Studies have also found that rotavirus related seizures could occur in both febrile (41%) and afebrile (59%) children [39, 40]. Therefore we recommend to do stool rotavirus antigen test in children who present with seizures and diarrhea. FS may be provoked by any febrile illness. Viral infections in particular, are frequently associated with FS. The prevalence of bacterial infections in children with FS is low, but such infections may be serious when they occur. In our study 25.4% of children had identified specific infections. We found these viruses; EBV, influenza A (IF A), parainfluenza (PIF) III & VIII, RSV, CMV and rotavirus (RV). However, not all known infectious agents were tested in our laboratory. Chung B et al found five viral agents were present in 34.4% of all admissions with FS in their 5 year study period; with the commonest five viruses being IF, adenovirus, PIF, RSV and RV [39]. In Asian countries, IF A is the most frequent cause [41], while in the United States, human herpesvirus (HHV)-6 infection is most commonly associated with FS and accounts for one third of all first-time cases in children up to 2 years of age [42]. Viruses were commonest infections by 65.3% like many other study findings. Atypical bacteria cases were also common in our study, mostly due to having many respiratory tract infections. Due to changes in viral susceptibility and seasonal variation, most cases of FS are reported to be in winter [26, 43].

FS includes fever in its definition, but up to present there is no cut point of fever level required to diagnose it. In most cases, height of body temperature is considered to play a more important role in pathogenesis of a FS than rapidity of the rise in temperature. In some studies lower maximum temperature during FS was found to be associated with recurrence of seizure and future epilepsy; and shorter interval between onset of fever and the initial seizure [4, 11]. However, in our study we found no statistical significance between recurrence and temperature. Some FS occur early, as the first feature of febrile illness in 25-50% of all cases [44]. Others occur after or during, but not necessarily at the peak level of fever. Most children in our study who had seizure after 12 hours since onset of fever had higher maximum temperature. This finding might be because these children had high threshold of seizure therefore it took time for the seizure to occur as a result, it happened when fever was higher. Viral infections were associated with higher temperature above 39°C in our study as they are known to cause fever that exceeds the individual threshold convulsive temperature [43].

### Limitations of the study

This was a retrospective study therefore there was no direct contact with the patient/caretaker to elaborate when more details were needed. Some findings were not significant due to the small study size. The details of single unprovoked seizures could not be specified due to lack of further investigations like inborn error of metabolism and lack of follow up. A prospective study will be helpful to express more regarding these problems.

## Conclusion

Seizures are the commonest neurological presentation in children worldwide and they bring fear and anxiety to the caretakers. From our study findings in Central China, most of childhood seizures are caused by FS resulting in good outcome and good prognosis like in Western countries. But we found out there are prolonged days of hospital stay which are not necessary. We suggest discharging these children early as they may acquire other nosocomial infections. Routine examination of brain imaging studies, blood sugar and electrolytes are not necessary. However, children with unprovoked seizures require brain imaging studies for better understanding of seizure etiology. Therefore we propose the attending clinician to decide carefully on which tests to order according to individual clinical presentation of each child to avoid excessive investigations which increase cost to the caretaker; avoid misuse of hospital resources, unnecessary panic and improper treatment.

### What is known about this topic

- Seizure is the commonest pediatric neurological disorder in pediatric population and is frightening to the caretakers;
- Febrile seizure is the commonest etiology of seizures in children and the prevalence is higher and poorer in tropical than western countries.

### What this study adds

- Febrile seizure was the commonest etiology of seizures by 87.5% and was mainly associated with viral infections;
- History of fever associated with seizures was common by 91% and in children aged 1 month to 5 years, while febrile seizures were common in children above 5 years;
- Children had prolonged days of hospitalization regardless of good prognosis and outcome.
